# Supplementary material for: A Comparative Study on Patient Safety Awareness Between Medical School Freshmen and Age-Matched Individuals
Source: Healthcare (Basel). 2024 Nov 14;12(22):2270. doi: 10.3390/healthcare12222270 (PMC11593893; doi:10.3390/healthcare12222270)
Supplement: Supplementary file 1 [file healthcare-12-02270-s001.zip › 20241105 Table S1_round2.pdf]

Table S1: Student responses to “perception” items regarding patient safety awareness, stratified by sex.

“Perception” items	Sex		Strongly Agree (%)	Agree (%)	Neutral (%)	Disagree (%)	Strongly Disagree (%)
5 Medical errors are inevitable	Male	Medical students	20.9	58.2	10.4	7.5	3.0
		Controls	7.6	62.1	22.7	4.5	3.0
	Female	Medical students	6.7	64.4	11.1	15.6	2.2
		Controls	11.5	53.8	24.4	10.3	0.0
6 Competent physicians do not make medical errors that lead to patient harm	Male	Medical students	3.0	11.9	17.9	41.8	25.4
		Controls	6.1	18.2	13.6	56.1	0.1
	Female	Medical students	0.0	8.9	24.4	51.1	15.6
		Controls	3.8	14.1	24.4	44.9	0.1
7 Medical errors can be eliminated through the efforts of physicians	Male	Medical students	1.5	43.3	20.9	25.4	9.0
		Controls	12.1	36.4	24.2	22.7	4.5
	Female	Medical students	0.0	37.8	33.3	26.7	2.2
		Controls	3.8	33.3	38.5	23.1	1.3
8 If I saw a medical error, I would report it to my supervisor	Male	Medical students	35.8	46.3	11.9	4.5	1.5
		Controls	13.6	42.4	24.2	16.7	3.0
	Female	Medical students	31.1	57.8	6.7	4.4	0.0
		Controls	12.8	47.4	24.4	14.1	1.3
9 If there is no harm to a patient, then there is no need to report medical errors to my supervisor	Male	Medical students	0.0	11.9	13.4	32.8	41.8
		Controls	4.5	13.6	13.6	45.5	22.7
	Female	Medical students	0.0	4.4	11.1	44.4	40.0
		Controls	0.0	5.1	10.3	46.2	38.5
10 If a medical error occurs because of my medical practice, I would always report it to my supervisor	Male	Medical students	38.8	46.3	11.9	3.0	0.0
		Controls	22.7	43.9	19.7	12.1	1.5
	Female	Medical students	51.1	46.7	0.0	2.2	0.0
		Controls	15.4	60.3	19.2	5.1	0.0
11 Establishing a system for reporting medical errors will lead to a reduction in the number of medical errors	Male	Medical students	37.3	43.3	9.0	10.4	0.0
		Controls	22.7	43.9	15.2	15.2	3.0
	Female	Medical students	35.6	46.7	8.9	8.9	0.0
		Controls	11.5	57.7	19.2	10.3	1.3
12 Working more carefully can effectively prevent the recurrence of similar medical errors	Male	Medical students	25.4	58.2	6.0	10.4	0.0
		Controls	24.2	54.5	13.6	7.6	0.0
	Female	Medical students	35.6	55.6	4.4	4.4	0.0
		Controls	25.6	69.2	3.8	1.3	0.0
13 Punishing the parties involved in medical errors does not reduce medical errors	Male	Medical students	11.9	41.8	25.4	17.9	3.0
		Controls	12.1	34.8	33.3	16.7	3.0
	Female	Medical students	4.4	37.8	35.6	20.0	2.2
		Controls	14.1	38.5	26.9	19.2	1.3
14 Increased safety awareness within hospitals would help reduce medical errors	Male	Medical students	35.8	59.7	4.5	0.0	0.0
		Controls	28.8	56.1	12.1	3.0	0.0
	Female	Medical students	42.2	57.8	0.0	0.0	0.0
		Controls	30.8	61.5	6.4	1.3	0.0
15 Healthcare professionals actively report medical errors to reduce the number of such errors	Male	Medical students	3.0	22.4	43.3	29.9	1.5
		Controls	3.0	13.6	39.4	36.4	7.6
	Female	Medical students	0.0	26.7	46.7	24.4	2.2
		Controls	2.6	7.7	51.3	34.6	3.8
