# Supplementary material for: A Comparative Study on Patient Safety Awareness Between Medical School Freshmen and Age-Matched Individuals
Source: Healthcare (Basel). 2024 Nov 14;12(22):2270. doi: 10.3390/healthcare12222270 (PMC11593893; doi:10.3390/healthcare12222270)
Supplement: Supplementary file 1 [file healthcare-12-02270-s001.zip › 20241105 Table S2_round2.pdf]

Table S2: Student responses to "knowledge" items regarding patient safety awareness, stratified by sex.

"Knowledge" items	Sex		Very good (%)	Good (%)	Fair (%)	Poor (%)	Very poor (%)
16 You know about "time out"	Male	Medical students	0.0	4.5	3.0	28.4	64.2
		Controls	6.1	6.1	10.6	16.7	60.6
	Female	Medical students	0.0	11.1	2.2	33.3	53.3
		Controls	2.6	9.0	3.8	14.1	70.5
17 You know about "hiyari-hatto*"	Male	Medical students	14.9	17.9	1.5	20.9	44.8
		Controls	1.5	6.1	9.1	7.6	75.8
	Female	Medical students	13.3	26.7	4.4	17.8	37.8
		Controls	0.0	9.0	1.3	7.7	82.1
18 You know about "double-check"	Male	Medical students	6.0	40.3	4.5	10.4	38.8
		Controls	1.5	16.7	12.1	13.6	56.1
	Female	Medical students	15.6	46.7	4.4	15.6	17.8
		Controls	6.4	25.6	10.3	11.5	46.2
19 You know about "Medical Accident Investigation System†"	Male	Medical students	4.5	13.4	1.5	25.4	55.2
		Controls	1.5	4.5	10.6	16.7	66.7
	Female	Medical students	4.4	26.7	4.4	26.7	37.8
		Controls	0.0	6.4	7.7	19.2	66.7
20 You know about "triage"	Male	Medical students	22.4	34.3	0.0	14.9	28.4
		Controls	9.1	15.2	9.1	10.6	56.1
	Female	Medical students	13.3	42.2	2.2	15.6	26.7
		Controls	9.0	10.3	2.6	2.6	75.6
21 You know about "team medicine"	Male	Medical students	22.4	70.1	6.0	0.0	1.5
		Controls	3.0	15.2	13.6	15.2	53.0
	Female	Medical students	35.6	60.0	4.4	0.0	0.0
		Controls	12.8	24.4	5.1	21.8	35.9
22 You are aware of an accident in which several patients died after undergoing laparoscopic surgery at a university hospital‡	Male	Medical students	11.9	40.3	0.0	25.4	22.4
		Controls	1.5	27.3	7.6	12.1	51.5
	Female	Medical students	17.8	46.7	4.4	15.6	15.6
		Controls	2.6	25.6	2.6	20.5	48.7
23 You know about "informed consent"	Male	Medical students	29.9	68.7	0.0	0.0	1.5
		Controls	15.2	51.5	6.1	4.5	22.7
	Female	Medical students	35.6	60.0	4.4	0.0	0.0
		Controls	12.8	61.5	7.7	6.4	11.5
24 You know about "evidence-based-medicine"	Male	Medical students	11.9	47.8	4.5	19.4	16.4
		Controls	1.5	9.1	12.1	16.7	60.6
	Female	Medical students	11.1	37.8	4.4	26.7	20.0
		Controls	1.3	5.1	3.8	12.8	76.9
25 Communication skills of healthcare professionals are relevant to medical errors	Male	Medical students	17.9	50.7	13.4	13.4	4.5
		Controls	3.0	18.2	15.2	25.8	37.9
	Female	Medical students	20.0	60.0	11.1	6.7	2.2
		Controls	5.1	25.6	12.8	23.1	33.3
