# Supplementary material for: A Comparative Study on Patient Safety Awareness Between Medical School Freshmen and Age-Matched Individuals
Source: Healthcare (Basel). 2024 Nov 14;12(22):2270. doi: 10.3390/healthcare12222270 (PMC11593893; doi:10.3390/healthcare12222270)
Supplement: Supplementary file 1 [file healthcare-12-02270-s001.zip › 20241105 Table S3_round2.pdf]

Table S3: Student responses to “attitude” items regarding patient safety awareness, stratified by sex.

“Attitude” items	Sex		Strongly Agree (%)	Agree (%)	Neutral (%)	Disagree (%)	Strongly Disagree (%)
26 Healthcare professionals should routinely spend part of their professional time in improving patient care	Male	Medical students	61.2	37.3	1.5	0.0	0.0
		Controls	34.8	51.5	7.6	1.5	4.5
	Female	Medical students	60.0	40.0	0.0	0.0	0.0
		Controls	41.0	52.6	6.4	0.0	0.0
27 “Patient safety” is an important topic in healthcare	Male	Medical students	70.1	29.9	0.0	0.0	0.0
		Controls	34.8	56.1	6.1	0.0	3.0
	Female	Medical students	62.2	35.6	2.2	0.0	0.0
		Controls	38.5	56.4	5.1	0.0	0.0
28 Learning about patient safety is important in medical universities and colleges	Male	Medical students	61.2	37.3	1.5	0.0	0.0
		Controls	33.3	47.0	16.7	0.0	3.0
	Female	Medical students	66.7	31.1	2.2	0.0	0.0
		Controls	47.4	48.7	3.8	0.0	0.0
29 You would like to learn more about “patient safety”	Male	Medical students	43.3	52.2	3.0	1.5	0.0
		Controls	6.1	22.7	40.9	16.7	13.6
	Female	Medical students	46.7	51.1	2.2	0.0	0.0
		Controls	10.3	28.2	35.9	14.1	11.5
30 You do not wish to support or advise a peer to decide how to respond to a medical error	Male	Medical students	0.0	4.5	22.4	46.3	26.9
		Controls	3.0	10.6	31.8	37.9	16.7
	Female	Medical students	4.4	2.2	8.9	57.8	26.7
		Controls	2.6	2.6	35.9	38.5	20.5
31 You want to analyze a case to find the cause of a medical error	Male	Medical students	46.3	52.2	1.5	0.0	0.0
		Controls	28.8	56.1	13.6	0.0	1.5
	Female	Medical students	48.9	48.9	2.2	0.0	0.0
		Controls	37.2	53.8	9.0	0.0	0.0
32 You will not disclose a medical error to the patient	Male	Medical students	3.0	22.4	28.4	38.8	7.5
		Controls	6.1	27.3	28.8	24.2	13.6
	Female	Medical students	2.2	15.6	42.2	24.4	15.6
		Controls	2.6	19.2	44.9	20.5	12.8
33 You will share all facts of the medical error with your colleagues to prevent recurrence	Male	Medical students	23.9	56.7	14.9	4.5	0.0
		Controls	19.7	54.5	21.2	3.0	1.5
	Female	Medical students	31.1	48.9	20.0	0.0	0.0
		Controls	24.4	44.9	28.2	2.6	0.0
